# Electrocardiogram-based mortality prediction in patients with COVID-19 using machine learning

**DOI:** 10.1007/s12471-022-01670-2

**Published:** 2022-03-17

**Authors:** R. R. van de Leur, H. Bleijendaal, K. Taha, T. Mast, J. M. I. H. Gho, M. Linschoten, B. van Rees, M. T. H. M. Henkens, S. Heymans, N. Sturkenboom, R. A. Tio, J. A. Offerhaus, W. L. Bor, M. Maarse, H. E. Haerkens-Arends, M. Z. H. Kolk, A. C. J. van der Lingen, J. J. Selder, E. E. Wierda, P. F. M. M. van Bergen, M. M. Winter, A. H. Zwinderman, P. A. Doevendans, P. van der Harst, Y. M. Pinto, F. W. Asselbergs, R. van Es, F. V. Y. Tjong, the CAPACITY-COVID collaborative consortium

**Affiliations:** 1 Department of Cardiology, Division of Heart and Lungs, University Medical Centre Utrecht, Utrecht University, Utrecht, The Netherlands; 2 Netherlands Heart Institute, Utrecht, The Netherlands; 3 Department of Clinical and Experimental Cardiology, Amsterdam University Medical Centres, Heart Center, Amsterdam Cardiovascular Sciences, University of Amsterdam, Amsterdam, The Netherlands; 4 Department of Clinical Epidemiology, Biostatistics & Bioinformatics, Amsterdam University Medical Centres, University of Amsterdam, Amsterdam, The Netherlands; 5 Department of Cardiology, Catharina Hospital Eindhoven, Eindhoven, The Netherlands; 6 Department of Cardiology, Jeroen Bosch Hospital, ’s-Hertogenbosch, The Netherlands; 7 Department of Cardiology, CARIM School for Cardiovascular Diseases, Maastricht University, Maastricht, The Netherlands; 8 Centre for Molecular and Vascular Biology, Department of Cardiovascular Sciences, KU Leuven, Leuven, Belgium; 9 Department of Cardiology, St. Antonius Hospital, Nieuwegein, The Netherlands; 10 Department of Cardiology, Amsterdam University Medical Centres, Amsterdam Cardiovascular Sciences, Vrije Universiteit Amsterdam, Amsterdam, The Netherlands; 11 Department of Cardiology, Dijklander Hospital, Hoorn, The Netherlands; 12 Central Military Hospital, Utrecht, The Netherlands; 13 Institute of Cardiovascular Science, Faculty of Population Health Sciences, University College London, London, UK; 14 Health Data Research UK and Institute of Health Informatics, University College London, London, UK

**Keywords:** COVID-19, Electrocardiogram, Machine learning, Deep learning, Arrhythmia, Mortality

## Abstract

**Background and purpose:**

The electrocardiogram (ECG) is frequently obtained in the work-up of COVID-19 patients. So far, no study has evaluated whether ECG-based machine learning models have added value to predict in-hospital mortality specifically in COVID-19 patients.

**Methods:**

Using data from the CAPACITY-COVID registry, we studied 882 patients admitted with COVID-19 across seven hospitals in the Netherlands. Raw format 12-lead ECGs recorded within 72 h of admission were studied. With data from five hospitals (*n* = 634), three models were developed: (a) a logistic regression baseline model using age and sex, (b) a least absolute shrinkage and selection operator (LASSO) model using age, sex and human annotated ECG features, and (c) a pre-trained deep neural network (DNN) using age, sex and the raw ECG waveforms. Data from two hospitals (*n* = 248) was used for external validation.

**Results:**

Performances for models a, b and c were comparable with an area under the receiver operating curve of 0.73 (95% confidence interval [CI] 0.65–0.79), 0.76 (95% CI 0.68–0.82) and 0.77 (95% CI 0.70–0.83) respectively. Predictors of mortality in the LASSO model were age, low QRS voltage, ST depression, premature atrial complexes, sex, increased ventricular rate, and right bundle branch block.

**Conclusion:**

This study shows that the ECG-based prediction models could be helpful for the initial risk stratification of patients diagnosed with COVID-19, and that several ECG abnormalities are associated with in-hospital all-cause mortality of COVID-19 patients. Moreover, this proof-of-principle study shows that the use of pre-trained DNNs for ECG analysis does not underperform compared with time-consuming manual annotation of ECG features.

**Supplementary Information:**

The online version of this article (10.1007/s12471-022-01670-2) contains supplementary material, which is available to authorized users.

## What’s new?

- An in-hospital all-cause mortality prediction model based on the 12-lead electrocardiogram (ECG) upon admission was developed and externally validated in a large cohort of COVID-19 patients.
- ECG-based prediction models might assist in risk stratification of COVID-19 patients, although with limited value above age and sex.
- Age, low QRS voltage, ST depression, premature atrial complexes, sex, increased ventricular rate, and right bundle branch block were predictors for mortality in COVID-19 patients.
- Transfer learning of a deep neural network to raw ECGs from a very heterogeneous source performed similarly to the time-consuming human interpretation of the ECG for mortality prediction in COVID-19 patients.

## Introduction

Since December 2019, Coronavirus Disease 2019 (COVID-19), spread around the world and caused a global pandemic. Several (cardiovascular) risk factors have been identified to be associated with increased morbidity and mortality [1]. Although vaccination has started in late 2020, the long-term protection of the vaccines is still unknown and the virus has already shown to mutate and a new variant could prove to be resistant to current vaccines [2]. Therefore, it is expected that COVID-19 will be a major ongoing burden to public health for the foreseeable future.

The electrocardiogram (ECG) is an easy to assess, widely available and inexpensive tool that is frequently used during the work-up of hospitalised COVID-19 patients. As the ECG can show signs of both cardiovascular risk factors, such as pathological Q‑waves, as well as current cardiac and pulmonary disease, it could be a useful tool for prognostication. Previous studies have already shown that individual ECG features—e.g. the presence of sinus tachycardia, atrial fibrillation, QRS prolongation, pathologic T‑wave inversion, non-specific repolarisation changes and signs of right ventricular strain at time of admission—yield value to predict in-hospital and out-of-hospital mortality in COVID-19 patients [3–5]. However, these studies only looked at individual risk factors and did not assess prediction models solely based on raw ECGs, a process that can be made autonomous without requiring human interaction.

Human interpretation of the ECG is a time-consuming task that suffers from high inter-observer variability [6]. Recently, machine learning and specifically the subclass of deep neural networks (DNNs) has gained much attention in the field of ECG analysis and has the advantage to learn complex patterns in the ECG signal, only based on the raw waveform as input. In short, DNNs can be described as algorithms using multiple layers which are able to gradually extract features from a raw input signal. For example, the first layers would extract only simple features, whilst later layers combine these into more complex and detailed features, which are then used for classification of a specific problem. This way both pattern discovery and classification are optimised jointly, which is a great advantage over the two-step approach when manually annotating ECGs [7]. Several studies have presented DNN models, analysing very large datasets of raw ECGs to diagnose a cardiac disease or to predict outcome of cardiac patients, often outperforming experienced cardiologists in these tasks [8–12]. Previous studies suggested that transfer learning, where a previously trained model is transferred to a new dataset or task, can improve performance of ECG-based DNNs [12, 13].

With this study, we aim to evaluate the value of using the ECG to predict in-hospital all-cause mortality of COVID-19 patients by specifically analysing the ECG at hospital admission. Furthermore, we aim to evaluate whether a transferred DNN can be trained on this new, relatively small multicentre dataset and complex task, in comparison with a logistic regression-based approach with manually interpreted ECG features and only age and sex. Secondly, we aim to identify specific ECG features associated with mortality in patients diagnosed with COVID-19.

## Methods

For this retrospective multicentre cohort study, we used data of seven hospitals in the Netherlands participating in the CAPACITY-COVID registry, which has been described previously [1, 14, 15]. In short, CAPACITY-COVID is an international consortium established to evaluate the role of cardiovascular disease in the COVID-19 pandemic. To date, data from 74 centres, originating from 13 different countries, leading to a dataset of >17,000 patient records. From a subset of patients included in the CAPACITY-COVID registry that were admitted to the emergency ward between March 1, 2020, and August 28, 2020, clinical data and raw-format 12-lead resting ECGs (XML) were collected. After collection, the ECGs were evaluated by multiple experienced investigators and ECG features were manually extracted, if present. Data was split into a development dataset and a validation dataset. Three mortality prediction models were developed: (a) a logistic regression baseline model using age and sex, (b) a least absolute shrinkage and selection operator (LASSO) model using age, sex and human annotated ECG features, and (c) a pre-trained deep neural network (DNN) using age, sex and the raw ECG waveforms. To better evaluate generalisability, we used data from two separate hospitals (*n* = 248) for external validation. A complete overview of the methods, including an extensive description of patient selection, data collection, ECG evaluation, data pre-processing and model development processes can be found in the Supplementary Methods section in the Electronic Supplementary Material (ESM).

## Results

From a total of 1396 COVID-19 patients from seven centres participating in the CAPACITY-COVID registry, 971 (69.5%) had a raw format ECG available, and 882 (63.2%) were found eligible for inclusion in this study (Fig. S1 in ESM). Baseline characteristics of the included patients are presented in Tab. 1, stratified by outcome. Overall, included patients had an age of 67 ± 14 years and 35% were female. Cardiovascular risk factors were common in this cohort, with hypertension in 51%, diabetes in 26%, and coronary artery disease in 21% of patients. Baseline characteristics, stratified per participating centre, are shown in Table S1 (in electronic supplementary material [ESM]). The final development dataset consisted of 634 patients, of which 179 (28%) died, and the validation dataset consisted of 248 patients, of which 57 (23%) died.

**Table 1:** Baseline characteristics of all patients included in this study stratified for mortality

	Overall	Survived	Died
*n*	882	646 (73)	236 (27)
Female sex (%)	309 (35)	243 (38)	66 (28)
Age (mean [SD])	67 (14)	64 (14)	75 (10)
BMI (mean [SD])	28 (5.3)	28 (5.3)	28 (5.2)
*History*			
Hypertension (%)	447 (51)	309 (48)	138 (59)
Diabetes (%)	230 (26)	153 (24)	77 (33)
Heart failure (%)	58 (6.6)	40 (6.2)	18 (7.6)
Coronary artery disease (%)	182 (21)	108 (17)	74 (31)
Valvular disease (%)	51 (5.8)	31 (4.8)	20 (8.5)
Supraventricular tachycardia (%)			
– Atrial flutter	13 (1.5)	7 (1.1)	6 (2.5)
– Paroxysmal AF	58 (6.6)	35 (5.4)	23 (9.7)
– Permanent AF	31 (3.5)	18 (2.8)	13 (5.5)
– Persistent AF	12 (1.4)	7 (1.1)	5 (2.1)
Ventricular tachycardia/fibrillation (%)			
– Non-sustained VT	4 (0.5)	4 (0.6)	0 (0.0)
– Sustained VT	6 (0.7)	4 (0.6)	2 (0.8)
– VF	5 (0.6)	2 (0.3)	3 (1.3)
*Medication at admission*			
Beta blocker (%)	262 (30)	165 (26)	97 (41)
Antiarrhythmic (%)	29 (3.3)	25 (3.9)	4 (1.7)
ACE (%)	164 (19)	112 (17)	52 (22)
ARB (%)	123 (14)	86 (13)	37 (16)
Diuretics (%)	214 (24)	138 (21)	76 (32)
*Current hospitalisation*			
LOS (median [IQR])	6 [3, 13]	6 [3, 13]	6 [4, 13]
Chloroquine (%)	446 (51)	300 (47)	146 (62)
Pulmonary embolism (%)	63 (7.1)	41 (6.3)	22 (9.3)
ICU (%)	198 (23)	123 (19)	75 (32)
Mechanical ventilation (%)	158 (18)	94 (15)	64 (27)

*AF* atrial fibrillation, *AV* atrioventricular, *ACE* angiotensin-converting enzyme, *ARB* angiotensin II receptor blocker, *BMI* body mass index, *ICU* intensive care unit, *IQR* interquartile range, *LOS* length of stay, *SD* standard deviation, *VF* ventricular fibrillation, *VT* ventricular tachycardia

### Baseline age and sex model

The baseline age and sex logistic regression model had a mean c‑statistic in the development dataset of 0.73 (95% confidence interval [CI] 0.69–0.77). In the validation dataset, the c‑statistic was 0.73 (95% CI 0.65–0.79). Age and female sex were both important predictors with coefficients of 0.90 (odds ratio [OR] 2.5 per one standard deviation [SD] or 14-year age increase) and −0.51 (OR 0.60) respectively.

### LASSO model

The manually derived ECG features used by the LASSO model are shown in Tab. 2, stratified by outcome. Of the 882 manually annotated ECGs, disagreement was resolved by consensus in a panel discussion in 74 (8.4%). The ventricular rate in the overall study was 90 ± 20 beats per minute, the majority was in sinus rhythm (85%), and one third had non-specific ST‑T abnormalities (34%). The final LASSO model consisted of 12 ECG features that were predictive for the outcome in the development dataset. The c‑statistic for the LASSO model was 0.76 (95% CI 0.72–0.80) in the development dataset and 0.76 (95% CI 0.69–0.82) in the validation dataset. The most important features for predicting mortality were age, low QRS voltage, ST depression, premature atrial complexes, ventricular rate and right bundle branch block (RBBB). Female sex and left bundle branch block were predictive for a lower risk of mortality. Coefficients of the ECG features included in the final model are shown in Fig. S2 (in ESM).

**Table 2:** ECG characteristics used in the logistic regression model, stratified for mortality

	Overall	Survived	Died
*N* (%)	882	646 (73)	236 (27)
Ventricular rate (mean [SD])	90 (20)	89 (18)	94 (23)
PR interval (median [IQR])	154 [139, 172]	154 [140, 170]	156 [138, 178]
QRS duration (median [IQR])	94 [86, 106]	93 [85, 106]	96 [86, 111]
QTc interval (median [IQR])	443 [423, 466]	441 [421, 463]	452 [427, 476]
*Rhythm*			
Primary rhythm (%)			
– Sinus rhythm	747 (85)	567 (88)	180 (76)
– Atrial rhythm	12 (1.4)	9 (1.4)	3 (1.3)
– Atrial fibrillation	114 (11)	66 (9.0)	48 (18)
– Paced	16 (1.8)	10 (1.5)	6 (2.5)
– Supraventricular tachycardia	7 (0.8)	2 (0.3)	5 (2.1)
– Other	10 (1.1)	8 (1.2)	2 (0.8)
PAC (%)	78 (7.8)	46 (6.3)	32 (11.9)
PVC (%)	69 (6.9)	38 (5.2)	31 (11.5)
*Conduction*			
1st degree AV block (%)	58 (6.6)	37 (5.7)	21 (8.9)
NICD (%)	35 (4.0)	17 (2.6)	18 (7.6)
LBBB (%)	27 (3.1)	23 (3.6)	4 (1.7)
RBBB (%)	64 (7.3)	38 (5.9)	26 (11.0)
Incomplete RBBB (%)	31 (3.5)	24 (3.7)	7 (3.0)
Long QT interval (%)	39 (4.4)	29 (4.5)	10 (4.2)
Left anterior fascicular block (%)	40 (4.5)	28 (4.3)	12 (5.1)
*Repolarisation*			
Aspecific ST-segment/T-wave abnormalities (%)	302 (34)	213 (33)	89 (37)
Pathologically negative Ts (%)	41 (4.6)	29 (4.5)	12 (5.1)
ST depression (%)	35 (4.0)	17 (2.6)	18 (7.6)
ST elevation (%)	16 (1.8)	9 (1.4)	7 (3.0)
*Other*			
Pathological Qs (%)	29 (3.3)	20 (3.1)	9 (3.8)
Pericarditis (%)	3 (0.3)	3 (0.5)	0 (0.0)
Low QRS voltages (%)	19 (2.2)	11 (1.7)	8 (3.4)
Left ventricular hypertrophy (%)	18 (2.0)	12 (1.9)	6 (2.5)
Clockwise rotation (%)	118 (13)	82 (13)	36 (15)
*Right heart strain*			
SIQIIITIII (%)	41 (4.6)	31 (4.8)	10 (4.2)
P pulmonale (%)	4 (0.5)	3 (0.5)	1 (0.4)
*Axis (%)*			
– Intermediate	650 (73.7)	493 (76.3)	157 (66.5)
– Left	170 (19.3)	111 (17.2)	59 (25.0)
– Right	62 (7.0)	42 (6.5)	20 (8.5)

*AV* atrioventricular, *LBBB* left bundle branch block, *NICD* nonspecific intraventricular conduction delay, *PAC* premature atrial complex, *PVC* premature ventricular complex, *RBBB* right bundle branch block, *SD* standard deviation

### Deep neural network

**Fig. 1:**
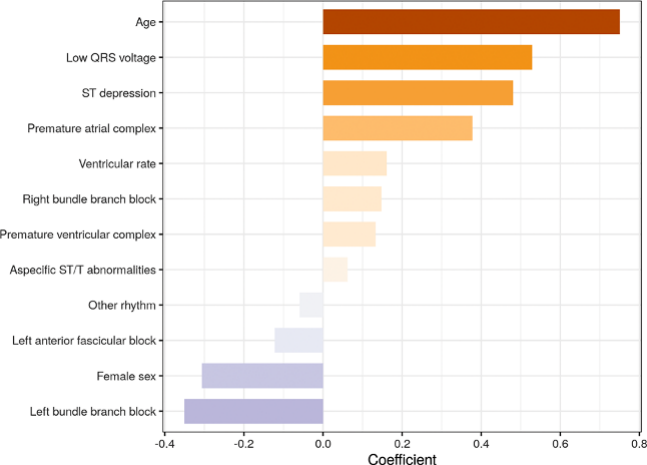
Variable importance for the manually extracted ECG features in the LASSO model. Coefficients represent the beta coefficients of the normalised variables in the LASSO model and can there be interpreted as importance values. Negative values point at a lower risk of mortality

**Fig. 2:**
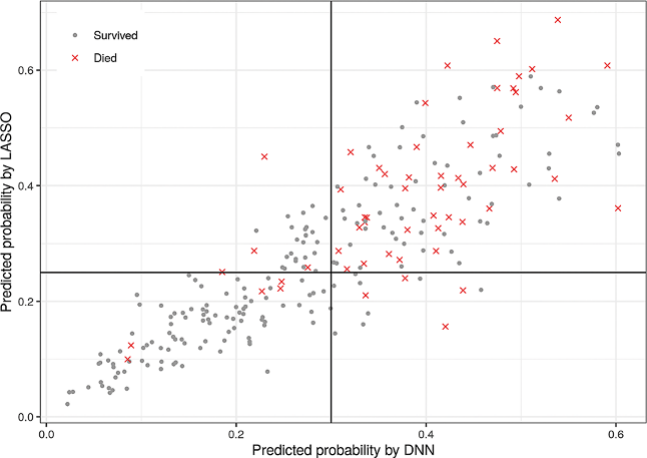
Predicted probability for the deep neural network compared with the LASSO algorithm with manually extracted ECG features, with the probability cut-offs of 30 and 25%, respectively. Inspection of the ECGs in the right lower corner (i.e. correct predictions by the DNN and not the LASSO) showed frequent tachycardia and low QRS voltage that did not meet the criteria, while the age was mostly below 70 years. Inspection of the left upper corner showed that these ECGs were normal, but patients had a high age of up to 93. (*DNN* deep neural network, *ECG* electrocardiogram, *LASSO* least absolute shrinkage and selection operator)

The c‑statistic for the deep neural network was 0.86 (95% CI 0.83–0.89) in the development dataset and 0.77 (95% CI 0.70–0.83) in the validation dataset. Calibration slope and intercept, sensitivity, specificity, positive predictive value (PPV), and negative predictive value (NPV) of all three methods for the validation dataset are presented in Tab. 3. An overview of both methods is shown in Fig. 1. Fig. 2 shows the differences in predicted probabilities between the DNN model and the LASSO model. Correlation between the two models was high (Pearson correlation *r* = 0.86, *p* < 0.001). Table S2 (in ESM) shows the performance for all three models in the different hospitals separately.

**Table 3:** Prognostic performance of the three models in the validation dataset

Measure	Baseline model	LASSO model	DNN model
C‑statistic	0.73 [0.65–0.79]	0.76 [0.68–0.82]	0.77 [0.70–0.83]
Calibration slope	1.02 [0.65–1.5]	1.32 [0.90–1.80]	1.46 [0.96–2.14]
Calibration intercept	−0.17 [−0.65–0.35]	−0.06 [−0.52–0.42]	−0.04 [−0.51–0.49]
Sensitivity	0.83	0.84	0.84
Specificity	0.52	0.56	0.63
Positive predictive value	0.34	0.36	0.41
Negative predictive value	0.90	0.92	0.93

Both measures of discriminatory and calibration performance are shown. Sensitivities, specificities, positive and negative predictive values are evaluated at probability cut-offs of 22, 25 and 30% respectively.

### Subgroup analyses

Performance was similar for males and females for all three models investigated. For males, c‑statistics ranged from 0.72 (95% CI 0.63–0.81) to 0.77 (95% CI 0.68–0.85) and 0.77 (95% CI 0.69–0.86) for the age and sex model, the LASSO model and the DNN respectively. For females, c‑statistics ranged from 0.74 (95% CI 0.63–0.85) to 0.76 (95% CI 0.65–0.86) and 0.76 (95% CI 0.65–0.86) respectively.

## Discussion

This study is the first to suggest that machine learning models are able to specifically predict in-hospital all-cause mortality in COVID-19 patients with the ECG during admission as input, although the added value to a model with only age and sex is limited. Two approaches were evaluated and externally validated in a unique multicentre cohort of COVID-19 patients: a classical LASSO logistic regression on human annotated ECG features and a fine-tuned DNN, which was previously pre-trained on raw ECG signals of almost 300,000 patients. This is one of the first studies to show that a pre-trained DNN can be easily transferred to other datasets, with other ECG machines and a completely different task, with similar accuracy as the very time-consuming manual extraction of ECG features. External validation was performed in different centres, which is uncommon in the current research on DNN-based ECG interpretation. This is a major finding, as it opens up possibilities for the highly dimensional DNNs in smaller datasets with complex prediction tasks. Finally, multiple new ECG abnormalities where detected that are predictive of mortality in COVID-19 patients.

In our dataset, we have demonstrated that the ECG alone, a simple and widely performed test, includes features with predictive value in risk stratification of COVID-19 patients. A previous large multicentre study has demonstrated that a prediction model based on clinical variables (such as age, sex, comorbidities, laboratory values and lab tests) performs well in predicting mortality in COVID-19 patients with c‑statistics up to 0.77 [16]. Our study suggests that the ECG performs similarly, with the advantage that it uses just one modality and can be automated. An important finding in the current study is the limited added predictive value above age and sex. This is in line with earlier studies that developed prediction models for in-hospital mortality and showed that, in multivariable models, prediction is mostly driven by age and sex. These studies did not evaluate age and sex alone, they included several other, mostly clinical, parameters, which might also have limited value above age and sex, similar to our findings [15–18]. Within our current study, we did not include any clinical data besides age and sex, because we aimed to evaluate only data derived from the ECG file itself. Age and sex are always present in the metadata of an ECG and therefore these could be included. The main reason for this was to use only one data modality, which is time efficient, instead of having to derive data from different sources, such as medical records or laboratory results.

The DNN used in the study, which was previously trained to perform comprehensive ECG interpretation on a large non-COVID-19 ECG dataset from four different countries, showed to be transferrable to the complex mortality prediction task of this study with minimal fine-tuning [13]. Even after external validation in different hospitals with different ECG devices, something which is rarely performed in machine learning studies, the transferred DNN remains of predictive value. It is especially important to note that while the DNN is pre-trained and fine-tuned on datasets mostly using GE MAC ECG devices, it also performs very well in external hospital 6, which uses the Welch Allyn Mortara device (Table S2 in ESM). Moreover, we observe that performance aligns with the human annotated model, which indicates that the ECG device (with different filters and electrodes) has very limited influence on the performance of DNNs. These are very promising findings, as it could allow the use of pre-trained ECG-DNNs for smaller datasets in the process of diagnosis or prediction using the raw format ECG. More conventional analysis techniques, such as multivariable logistical regression, require manually selected features as input, for which collection and labelling of these features is a time-consuming process when analysing large datasets. In our study, the performance of our deep learning model was similar to that of the LASSO logistic regression, illustrating the potential of deep learning as a time-efficient raw ECG data analysis technique. This is especially important in settings where expert knowledge in ECG interpretation is not readily available. Although the performance of the DNN model was still similar to the performance of the logistic regression model in the external validation set, the c‑statistic did decrease from 0.86 to 0.77. This indicates that the DNN model, despite measures taken to prevent overfitting (i.e. pre-training, freezing of layers and dropout), is still overconfident in the training dataset due to the larger number of trainable parameters. It remains important to use rigorous validation methods in deep learning research to obtain reliable estimates of predictive performance.

The current evaluation of ECG features in 882 COVID-19 patients is the largest to date, containing data collected from seven different hospitals across the Netherlands [1, 15]. Previous studies have described ECG features associated with poor outcome of COVID-19 patients. For example, Raad et al. identified ECG abnormalities associated with right ventricular strain (such as SIQIIITIII pattern and incomplete/complete RBBB) to be associated with the need for mechanical ventilation and mortality in patients admitted with COVID-19 [4]. With our data, we reproduced that the presence of RBBB is predictive of in-hospital mortality, but we were not able to do the same for the other features of right ventricular strain. A possible explanation for this is that the patients included in the study of Raad et al. were more severely affected by COVID-19, e.g. right ventricular overload due to severe pulmonary disease and potentially pulmonary embolisms, than the patients in our study [4]. Another study by McCullough et al. evaluating 756 COVID-19 patients, showed the presence of premature atrial contractions, RBBB and ischaemic T‑wave inversion to be associated with mortality, which corresponds to our findings [3]. This study is the first to identify ST depression and low QRS voltages as risk factors for mortality in COVID-19 patients. While ST depression may be a sign of myocardial hypoxia due to malperfusion, low QRS voltages may either represent emphysema and obesity, which are both previously identified as important risk factors for adverse events in COVID-19 patients. Low QRS voltages may also represent pericardial effusion, although pericarditis was only diagnosed in one patient in the entire CAPACITY cohort [1, 15].

### Limitations

There are several limitations to acknowledge. No follow-up data was available to us after patients were discharged from the hospital, therefore we could only assess the in-hospital mortality and mortality after discharge was not included in this study. The multicentric nature of this study is a strength. However, since not all hospitals included in this study use the same ECG machine and different machines use different filtering settings. This could possibly have introduced bias when analysing the raw ECG data using deep learning. To minimise this effect, we applied several pre-processing steps to normalise the input data per hospital. Furthermore, it is difficult to understand what features DNNs learn for their prediction and also in this study, we do not know the ECG features used by the DNN. Some techniques have been developed for this purpose, however visualisation of DNN remains a topic of ongoing research and therefore could be considered in near future. Finally, the models were evaluated in an external dataset with two Dutch hospitals, which might limit the generalisability to other countries or settings.

## Conclusion

This study shows that ECG-based prediction models could be helpful for initial risk stratification of patients diagnosed with COVID-19, although the added value above age and sex is limited. Secondarily, several ECG abnormalities are associated with in-hospital all-cause mortality of COVID-19 patients. Furthermore, this proof-of-principle study shows that the use of pre-trained DNNs for ECG analysis does not underperform compared with multivariable regression analysis of ECG features derived using time-consuming human interpretation.

## Supplementary Information

Supplementary material including additional methods, results, figures and tables

**Fig. S1** Flow chart of the patient selection process. (*ECG* electrocardiogram, *ICU* Intensive Care Unit)

**Fig. S2** Overview of the two machine learning methods used. The LASSO approach consists of two steps, where the ECGs are (1) manually evaluated by a panel of physicians and (2) classified, while the deep neural networks takes raw ECGs as input and classifies in an end-to-end manner. This specific case had a high probability of in-hospital mortality. Follow-up of this case showed that the patient had died during admission. (*ECG* electrocardiogram, *LASSO* least absolute shrinkage and selection operator)
