# Supplementary material for: Electrocardiogram-based mortality prediction in patients with COVID-19 using machine learning
Source: Neth Heart J. 2022 Mar 17;30(6):312–8. doi: 10.1007/s12471-022-01670-2 (PMC8929464; doi:10.1007/s12471-022-01670-2)
Supplement: Supplementary file 1 — Supplementary material including additional methods, results, figures and tables [file 12471_2022_1670_MOESM1_ESM.docx]

Supplementary methods

*Study participants*

For this retrospective multicentre cohort study, data from the CAPACITY-COVID registry was used of seven participating hospitals in the Netherlands.(1–3) Patients admitted to one of these seven centres between March 1, 2020 and August 28, 2020 were included if a baseline 12-lead ECG was performed within 72 hours of primary admission to the emergency ward. Patients were excluded when they were directly transferred to the Intensive Care Unit (ICU) from another hospital, because our main focus was to predict outcome in patients primarily presented at the emergency ward. ECGs were assessed for technical adequacy and were excluded in case of limb lead reversal, loss of signal on one or more leads, or when a high amount of noise on two or more leads was present. ECGs acquired after chloroquine or hydrochloroquine administration were excluded. COVID-19 was diagnosed if a patient had a positive SARS-CoV-2 Polymerase Chain Reaction (PCR). The primary outcome for the prediction model was in-hospital all-cause mortality (hereafter mortality) within 30 days after admission. Local ethics approval was obtained in all participating hospitals. The informed consent procedure is described in detail elsewhere.(2,3)

*Data collection*

Data was collected as part of the CAPACITY-COVID registry, and a specification of all collected variables included can be found in a recent publication on the CAPACITY-COVID registry and on the CAPACITY-COVID website ([www.capacity-covid.eu](http://www.capacity-covid.eu)).(2,3) For this study, pseudonymised raw-format 12-lead resting ECGs of all the enrolled study participants, either in XML or DICOM format, were used. The ECGs included in this study were obtained by ECG machines from different manufacturers (General Electric MAC5500, Welch Allyn Mortara ELI-250 and ELI-280, Philips PageWriter TC70, and Philips IntelliVue) and were recorded at either 250, 500, or 1000 Hz.

*Evaluation of 12-lead electrocardiograms*

After collection, the 12-lead ECGs were visualised in a standardised way and evaluated by six investigators with clinical experience in ECG assessment (R.v.d.L., H.B., K.T., J.G., T.M., and F.T.). A full ECG interpretation, based on the American Heart Association’s Electrocardiography Diagnostic Statement List(4), was performed using an online tool and included extra markers for right heart strain (clockwise rotation, SIQIIITIII pattern and P-pulmonale).(5) Each ECG was assessed by two separate annotators, who were blinded for clinical (outcome) data and for assessment by the other annotator. Any disagreement in evaluation between investigators was discussed by the annotating committee until consensus was reached. Heart rate and conduction intervals were calculated by the algorithms specific to every ECG machine and were visible to the annotators.

*Data pre-processing and development of machine learning models*

Prognostic prediction models were developed using data from five of the seven participating centres (*development dataset*), while the data from the remaining two centres (one large academic hospital and one large general hospital) were used for external validation (*validation dataset*). Training and hyperparameter optimisation was performed in the development dataset, while the validation or test dataset was used as a hold-out set with unseen data to determine the generalisability of the models. All ECG waveforms were converted to millivolts and resampled to 500 Hz using linear interpolation, lead III, aVF, aVL and aVR were derived from lead I and II. A moving median filter with a window of 600ms was used to minimise baseline wander.

Firstly, a baseline logistic regression model was developed using age and sex as predictors. Secondly, in a least absolute shrinkage and selection operator (LASSO) logistic regression model, age, sex and the manually annotated ECG features from the *development dataset* were used.(6) All continuous variables were normalised and 10-fold cross-validation was used in the development dataset to determine an optimal lambda value. Importance of the features was assessed with the beta coefficients. Thirdly, a deep learning model was trained on the raw 12-lead waveforms from all patients in the *development dataset*. A model that was previously developed as part of the 2020 Physionet/Computing in Cardiology Challenge was used, which is described in more detail previously.(7) This ﻿exponentially dilated causal convolutional neural network was trained on 254.044 ECGs from the University Medical Center Utrecht and fine-tuned on 41.511 ECGs from five different countries to predict the 24 most common ECG diagnoses with c-statistics between 0.751 and 0.992. This pre-trained model was fine-tuned for the current prediction task with all but the last linear layer frozen to prevent overfitting. This last layer was adjusted to only one outcome (instead of the previously used 24) and age and sex were added. Training was performed using the Adam optimiser with a learning rate of 0.001 and a batch size of 16.(7) As most of the model was frozen, no further hyperparameter optimisation was performed. Data pre-processing and development of machine- and deep learning models was performed using Python version 3.7, PyTorch version 1.7, and R version 3.6.3.

*Statistical analysis*

Normality of the baseline and ECG variables was assessed visually by the use of histograms and QQ-plots. Numerical values were presented as median [IQR] or mean ± standard deviation, where appropriate. Categorical variables are displayed as absolute value and percentages. There were no missing data in the predictors or outcome.

Performance of the prediction models (i.e. discrimination and calibration) was assessed using the c-statistic (equivalent to the area under the receiver operating curve, AUC) and calibration intercept and slope. For the external validation datasets, c-statistics, calibration intercepts and slopes, sensitivity, specificity, positive predictive value (PPV), and negative predictive value (NPV) were estimated. As a rule-out algorithm is clinically most relevant, sensitivity, specificity, PPV, and NPV were evaluated in the validation set using a probability threshold that achieved a sensitivity of approximately 85%. Considering the small number of centres or clusters, bootstrapping of individual data points was performed to estimate the 95% confidence intervals using 2000 bootstrap samples.(8) All statistical analyses were performed using R version 3.6.3, and the TRIPOD guidelines for transparent reporting of multivariable prediction models were adhered to throughout the manuscript.(9)

*Reproducibility and Open Access*

Supplementary references

The data used in this study is not publicly available. The code is available upon request.

1. Linschoten And M, Asselbergs FW. CAPACITY-COVID: a European Registry to determine the role of cardiovascular disease in the COVID-19 pandemic. Eur Heart J. 2020;

2. Linschoten M, Peters S, van Smeden M, Jewbali LS, Schaap J, Siebelink H-M, et al. Cardiac complications in patients hospitalised with COVID-19. Eur Hear J Acute Cardiovasc Care. 2020;

3. Linschoten M, Asselbergs FW. Clinical presentation, disease course and outcome of COVID-19 in hospitalized patients with and without pre-existing cardiac disease – a cohort study across sixteen countries. medRxiv. 2021;

4. Mason JW, Hancock EW, Gettes LS. Recommendations for the standardization and interpretation of the electrocardiogram: Part II: Electrocardiography diagnostic statement list: A scientific statement from the American Heart Association Electrocardiography and Arrhythmias Committee, Council . Circulation. 2007.

5. Stein PD, Matta F, Sabra MJ, Treadaway B, Vijapura C, Warren R, et al. Relation of electrocardiographic changes in pulmonary embolism to right ventricular enlargement. Am J Cardiol. 2013;

6. Tibshirani R. Regression Shrinkage and Selection Via the Lasso. J R Stat Soc Ser B. 1996;

7. Bos MN, van de Leur RR, Vranken JF, Gupta DK, van der Harst P, Doevendans PA, et al. Automated Comprehensive Interpretation of 12-lead Electrocardiograms Using Pre-trained Exponentially Dilated Causal Convolutional Neural Networks. Comput Cardiol (2010). 2020;47.

8. Bouwmeester W, Moons KGM, Kappen TH, Van Klei WA, Twisk JWR, Eijkemans MJC, et al. Internal validation of risk models in clustered data: A comparison of bootstrap schemes. Am J Epidemiol. 2013;

9. Moons KGM, Altman DG, Reitsma JB, Ioannidis JPA, Macaskill P, Steyerberg EW, et al. Transparent reporting of a multivariable prediction model for individual prognosis or diagnosis (TRIPOD): Explanation and elaboration. Ann Intern Med. 2015;

*Table S1. Baseline characteristics per participating centre.*

|  | Training data | | | | | Validation data | |
| --- | --- | --- | --- | --- | --- | --- | --- |
|  | 1 | 2 | 3 | 4 | 5 | Academic hospital | General hospital |
| n | 158 | 70 | 165 | 221 | 20 | 95 | 153 |
| Female sex (%) | 64 (40.5) | 27 (38.6) | 45 (27.3) | 65 (29.4) | 8 (40.0) | 39 (41.1) | 61 (39.9) |
| Age (mean (SD)) | 60.97 (14.69) | 64.00 (15.02) | 70.59 (13.83) | 70.67 (11.12) | 68.95 (11.34) | 63.09 (15.24) | 67.86 (12.91) |
| BMI (mean (SD)) | 28.50 (5.63) | 27.46 (5.54) | 27.16 (4.02) | 27.93 (4.88) | 26.39 (5.59) | 27.12 (5.40) | 29.00 (6.05) |
| **History** |  |  |  |  |  |  |  |
| Cardiovascular disease (%) | 47 (30.3) | 34 (48.6) | 71 (43.0) | 86 (38.9) | 6 (31.6) | 19 (20.0) | 60 (39.2) |
| Hypertension (%) | 83 (52.5) | 36 (51.4) | 76 (46.1) | 108 (48.9) | 8 (40.0) | 40 (42.1) | 96 (62.7) |
| Diabetes (%) | 42 (26.6) | 21 (30.0) | 29 (17.6) | 59 (26.7) | 2 (10.0) | 22 (23.2) | 55 (35.9) |
| Heart failure (%) | 9 (5.7) | 8 (11.4) | 11 (6.7) | 15 (6.8) | 1 (5.0) | 4 (4.2) | 10 (6.5) |
| Coronary artery disease (%) | 27 (17.1) | 17 (24.3) | 40 (24.2) | 50 (22.6) | 3 (15.0) | 10 (10.5) | 35 (22.9) |
| Valvular disease (%) | 7 (4.4) | 6 (8.6) | 13 (7.9) | 11 (5.0) | 1 (5.0) | 1 (1.1) | 12 (7.8) |
| Supraventricular tachycardia (%) |  |  |  |  |  |  |  |
| Atrial flutter | 2 (1.3) | 1 (1.4) | 3 (1.8) | 3 (1.4) | 0 (0.0) | 0 (0.0) | 4 (2.6) |
| Paroxysmal AF | 5 (3.2) | 10 (14.3) | 16 (9.7) | 13 (5.9) | 0 (0.0) | 4 (4.2) | 10 (6.5) |
| Permanent AF | 1 (0.6) | 1 (1.4) | 9 (5.5) | 10 (4.5) | 4 (20.0) | 4 (4.2) | 2 (1.3) |
| Persistent AF | 5 (3.2) | 0 (0.0) | 2 (1.2) | 4 (1.8) | 0 (0.0) | 0 (0.0) | 1 (0.7) |
| Ventricular tachycardia/fibrillation (%) |  |  |  |  |  |  |  |
| Non-sustained VT | 0 (0.0) | 0 (0.0) | 1 (0.6) | 0 (0.0) | 0 (0.0) | 1 (1.1) | 2 (1.3) |
| sustained VT | 1 (0.6) | 0 (0.0) | 1 (0.6) | 1 (0.5) | 0 (0.0) | 1 (1.1) | 2 (1.3) |
| VF | 0 (0.0) | 0 (0.0) | 1 (0.6) | 3 (1.4) | 0 (0.0) | 1 (1.1) | 0 (0.0) |
| **Medication history** |  |  |  |  |  |  |  |
| Beta blocker (%) | 43 (27.2) | 25 (35.7) | 54 (32.7) | 73 (33.0) | 4 (20.0) | 19 (20.0) | 44 (28.8) |
| Antiarrhythmic (%) | 5 (3.2) | 0 (0.0) | 4 (2.4) | 6 (2.7) | 0 (0.0) | 2 (2.1) | 12 (7.8) |
| ACE (%) | 29 (18.4) | 22 (31.4) | 27 (16.4) | 36 (16.3) | 3 (15.0) | 11 (11.6) | 36 (23.5) |
| ARB (%) | 13 (8.2) | 8 (11.4) | 26 (15.8) | 45 (20.4) | 3 (15.0) | 10 (10.5) | 18 (11.8) |
| Diuretics (%) | 36 (22.8) | 15 (21.4) | 39 (23.6) | 65 (29.4) | 2 (10.0) | 15 (15.8) | 42 (27.5) |
| **Current hospitalisation** |  |  |  |  |  |  |  |
| LOS (median [IQR]) | 5.00 [3.00, 10.00] | 9.00 [5.00, 16.25] | 7.00 [3.00, 14.00] | 6.00 [4.00, 14.00] | 6.00 [3.00, 18.00] | 5.00 [3.00, 11.00] | 6.50 [3.00, 13.00] |
| Chloroquine (%) | 3 (1.9) | 56 (80.0) | 126 (76.4) | 161 (72.9) | 5 (25.0) | 1 (1.1) | 94 (61.4) |
| Pulmonary embolism (%) | 10 (6.3) | 7 (10.0) | 6 (3.6) | 22 (10.0) | 1 (5.0) | 8 (8.4) | 9 (5.9) |
| ICU (%) | 31 (19.6) | 19 (27.1) | 38 (23.0) | 60 (27.1) | 8 (40.0) | 19 (20.0) | 23 (15.0) |
| Mechanical ventilation (%) | 27 (17.1) | 15 (21.4) | 29 (17.6) | 47 (21.3) | 8 (40.0) | 13 (13.7) | 19 (12.4) |
| Mortality (%) | 27 (17.1) | 22 (31.4) | 51 (30.9) | 76 (34.4) | 3 (15) | 13 (13.7) | 22 (31.4) |

*AF: atrial fibrillation, AV: atrioventricular, ACE: angiotensin converting enzyme, ARB: angiotensin II receptor blocker, ICU: intensive care unit, LOS: length of stay, VF: ventricular fibrillation, VT: ventricular tachycardia.*

*Table S2. Performance of the three models for the hospitals included separately. The first 5 hospitals were used for training of the models, while hospitals 6 and 7 were used for validation.*

| **Center** | **Type** | **ECG device** | **Died**  **(n (%))** | **Total (n)** | **C-statistic** | | |
| --- | --- | --- | --- | --- | --- | --- | --- |
|  |  |  |  |  | **AS** | **ASECG** | **DNN** |
| 1 | Academic | GE MAC5500 | 27 (17) | 158 | 0.70 | 0.72 | 0.84 |
| 2 | Academic | GE MAC5500 | 22 (31) | 70 | 0.64 | 0.71 | 0.79 |
| 3 | General | GE MAC5500 | 51 (31) | 165 | 0.73 | 0.76 | 0.87 |
| 4 | Academic | GE MAC5500 | 76 (34) | 221 | 0.77 | 0.78 | 0.89 |
| 5 | General | Welch Allyn Mortara ELI-250 | 3 (15) | 30 | 0.45 | 0.69 | 0.69 |
| 6 | Academic | Welch Allyn Mortara ELI-280 | 13 (14) | 95 | 0.78 | 0.83 | 0.87 |
| 7 | General | Philips PageWriter TC70 | 44 (29) | 153 | 0.69 | 0.71 | 0.72 |

**Collaborators within the CAPACITY-COVID consortium (listed alphabetically)**

Al-Ali AK^1^, Al-Muhanna FA^2^, Al-Windy NYY^3^, Almubarak YA^4^, Alnafie AN^5^, Alshahrani M^6^, Alshehri AM^7^, Anthonio RL^8^, Asselbergs FW^9,10,11^, Aujayeb A^12^, ten Berg JM^13^, van Boxem AJM^14^, Captur G^11,15^, Caputo M^16,17^, Charlotte N^18^, Dark P^19^, De Sutter J^20,21^, Delsing CE^22^, Dorman HGR^23^, Drost JT^24^, Emans ME^25^, Ferreira JB^26^, Gabriel L^27^, van Gilst WH^28^, Groenemeijer BE^29^, Haerkens-Arends HE^30^, van der Harst P^9^, Hedayat B^31^, van der Heijden DJ^32^, Hellou E^33^, Hermanides RS^34^, Hermans-van Ast JF^35^, van Hessen MWJ^36^, Heymans SRB^37,38,39^, van der Horst ICC^40,41^, van Ierssel SH^42^, Jewbali LS^43,44^, Kearney MT^45^, van Kesteren HAM^46^, Kietselaer BLJH^47^, Koning AMH^48^, Kopylov PY^49^, Kuijper AFM^50^, Kwakkel-van Erp JM^51^, van der Linden MMJM^52^, Linschoten M^9^, Linssen GCM^53^, Macias Ruiz R^54^, Magdelijns FJH^55^, Martens FMAC^56^, McCann GP^57^, van der Meer P^58^, Meijs MFL^59^, Messiaen P^60,61^, Monraats PS^62^, Montagna L^63^, Moriarty A^64^, Mosterd A^65^, Nierop PR^66^, van Ofwegen-Hanekamp CEE^67^, Pinto YM^68^, Poorhosseini H^69^, Prasad S^70,71^, Redón J^72,73^, Reidinga AC^74^, Ribeiro MIA^75^, Ripley DP^76^, Salah R^77^, Saneei E^78^, Saxena M^79^, Schaap J^80,81^, Schellings DAAM^82^, Schut A^80^, Shafiee A^83^, Shore AC^84^, Siebelink HJ^85^, van Smeden M^86^, Smits PC^87^, Pisters R^88^, Tessitore E^89^, Tieleman RG^28,90^, Timmermans P Jr^91^, Tio RA^92,93^, Tjong FVY^68,94,95^, den Uil CA^43,44,96^, Van Craenenbroeck EM^97^, van Veen HPAA^98^, Veneman T^99^, Verschure DO^100^, de Vries JK^101^, van de Wal RMA^102^, van de Watering DJ^103^, Westendorp ICD^104^, Westendorp PHM^105^, Weytjens C^106^, Wierda E^95^, Williams B^107^, Woudstra P^108^, Wu KW^109^, Zaal R^110^, Zaman AG^111^, van der Zee PM^112^

1. Department of Clinical Biochemistry, King Fahd Hospital of the University, Imam Abdulrahman Bin Faisal University, Alkhobar, Saudi Arabia
2. Department of Internal Medicine, King Fahd Hospital of the University, Imam Abdulrahman Bin Faisal University, Alkhobar, Saudi Arabia
3. Department of Cardiology, Gelre Hospital Zutphen, Zutphen, the Netherlands
4. Department of Critical Care, King Fahd Hospital of the University, Imam Abdulrahman Bin Faisal University, Alkhobar, Saudi Arabia
5. Department of Pathology, King Fahd Hospital of the University, Imam Abdulrahman Bin Faisal University, Alkhobar, Saudi Arabia
6. Department of Emergency Medicine, King Fahd Hospital of the University, Imam Abdulrahman Bin Faisal University, Alkhobar, Saudi Arabia
7. Department of Internal Medicine, Cardiology Section, King Fahd Hospital of the University, Imam Abdulrahman Bin Faisal University, Alkhobar, Saudi Arabia
8. Department of Cardiology, Treant Zorggroep, Emmen, the Netherlands
9. Department of Cardiology, Division of Heart and Lungs, University Medical Center Utrecht, Utrecht University, Utrecht, the Netherlands
10. Health Data Research United Kingdom and Institute of Health Informatics, University College London, London, United Kingdom
11. Institute of Cardiovascular Science, Faculty of Population Health Sciences, University College London, London, United Kingdom
12. Department of Respiratory and Acute Medicine, Northumbria Healthcare NHS Foundation Trust, Newcastle, United Kingdom
13. Department of Cardiology, St. Antonius Hospital, Nieuwegein, the Netherlands
14. Department of Pulmonology, Bravis Hospital, Roosendaal, the Netherlands
15. Department of Cardiology, Royal Free London NHS Foundation Trust, London, United Kingdo
16. Bristol Heart Institute, University Hospitals Bristol and Weston NHS Foundation Trust, Bristol, United Kingdom
17. Bristol Medical School, University of Bristol, Bristol, United Kingdom
18. Department of Cardiology, SSR Val Rosay, Saint Didier au Mont d'Or, Franc
19. Department of Critical Care, Salford Royal NHS Foundation Trust, Salford, United Kingdom
20. Department of Cardiology, AZ Maria Middelares, Ghent, Belgium
21. Department of Internal Medicine, Ghent University, Ghent, Belgium
22. Department of Internal Medicine and Infectious Diseases, Medisch Spectrum Twente, Enschede, the Netherlands
23. Department of Cardiology, Bravis Hospital, Roosendaal, the Netherlands
24. Department of Cardiology, Saxenburgh Medical Center, Hardenberg, the Netherlands
25. Department of Cardiology, Ikazia Hospital, Rotterdam, the Netherlands
26. Department of Cardiology, Hospital Professor Doutor Fernando Fonseca, Amadora, Portugal
27. Department of Cardiology, CHU UCL Namur site Godinne, Université Catholique de Louvain, Yvoir, Belgium
28. Department of Cardiology, University Medical Center Groningen, Groningen, the Netherlands
29. Department of Cardiology, Gelre Hospital Apeldoorn, Apeldoorn, the Netherlands
30. Department of Cardiology, Jeroen Bosch Hospital, 's-Hertogenbosch, the Netherlands
31. Department of Cardiology, Tehran Heart Center, Cardiovascular Diseases Research Institute, Tehran University of Medical Sciences, Tehran, Iran
32. Department of Cardiology, Haaglanden Medical Center, the Hague, the Netherlands
33. Department of Cardiology, E.M.M.S Hospital, Nazareth, Israel
34. Department of Cardiology, Isala Hospital, Zwolle, the Netherlands
35. Durrer Center, Netherlands Heart Institute, Utrecht, the Netherlands
36. Department of Cardiology, Groene Hart Hospital, Gouda, the Netherlands
37. Department of Cardiology, Cardiovascular Research Institute Maastricht (CARIM), Maastricht University Medical Center+, Maastricht, the Netherlands
38. Department of Cardiovascular Sciences, Center for Molecular and Vascular Biology, KU Leuven, Belgium
39. The Netherlands Heart Institute, Utrecht, the Netherlands
40. Department of Intensive Care, Maastricht University Medical Center+, Maastricht University, Maastricht, the Netherlands
41. Cardiovascular Research Institute Maastricht (CARIM), Maastricht University Medical Center, Maastricht, the Netherlands
42. Department of General Internal Medicine, Infectious Diseases and Tropical Medicine, Antwerp University Hospital, Antwerp, Belgium
43. Department of Cardiology, Erasmus MC University Medical Center, Rotterdam, the Netherlands
44. Department of Intensive Care, Erasmus MC University Medical Center, Rotterdam, the Netherlands
45. Leeds Institute for Cardiovascular and Metabolic Medicine, University of Leeds, Leeds, United Kingdom
46. Department of Cardiology, Admiraal de Ruyter Hospital, Goes, the Netherlands
47. Department of Cardiology, Zuyderland Medical Center, Heerlen, the Netherlands
48. Department of Gynaecology, Amstelland Hospital, Amstelveen, the Netherlands
49. World-Class Research Center Digital Biodesign and Personalized Healthcare, I.M. Sechenov First Moscow State Medical University, Sechenov University, Moscow, Russia
50. Department of Cardiology, Spaarne Gasthuis, Haarlem, the Netherlands
51. Department of Pulmonology, Antwerp University Hospital, University of Antwerp, Edegem, Belgium
52. Department of Cardiology, Franciscus Vlietland, Schiedam, the Netherlands
53. Department of Cardiology, Ziekenhuis Groep Twente (ZGT), Almelo, the Netherlands
54. Arrhythmias Unit, Department of Cardiology, Hospital Universitario Virgen de las Nieves, Granada, Spain
55. Department of Internal Medicine, Division of General Internal Medicine, Section Geriatric Medicine, Cardiovascular Research Institute Maastricht (CARIM), Maastricht University Medical Center+, Maastricht, the Netherlands
56. Department of Cardiology, Deventer Hospital, Deventer, the Netherlands
57. Department of Cardiovascular Sciences, University of Leicester and Cardiovascular Theme, National Institute for Health Research (NIHR) Leicester Biomedical Research Center, Glenfield Hospital, Leicester, United Kingdom
58. Department of Cardiology, LangeLand Hospital, Zoetermeer, the Netherlands
59. Department of Cardiology, Thorax Center Twente, Medisch Spectrum Twente, Enschede, the Netherlands
60. Department of Infectious Diseases & Immunity, Jessa Hospital, Hasselt, Belgium
61. Faculty of Medicine and Life Sciences, Hasselt University, Hasselt, Belgium
62. Department of Cardiology, Elizabeth-TweeSteden Hospital, Tilburg, the Netherlands
63. Department of Cardiology, A.O.U. San Luigi Gonzaga, Orbassano, Turin, Italy
64. Cardiovascular Research Unit, Craigavon Area Hospital, Southern Health and Social Care Trust, Portadown, Nothern Ireland
65. Department of Cardiology, Meander Medical Center, Amersfoort, the Netherlands
66. Department of Cardiology, Franciscus Gasthuis, Rotterdam, the Netherlands
67. Department of Cardiology, Diakonessenhuis, Utrecht, the Netherlands
68. Amsterdam University Medical Center, University of Amsterdam, Heart Center; Department of Clinical and Experimental Cardiology, Amsterdam Cardiovascular Sciences, Amsterdam, the Netherlands
69. Department of Interventional Cardiology, Tehran Heart Center, Cardiovascular Diseases Research Institute, Tehran University of Medical Sciences, Tehran, Iran
70. National Heart and Lung Institute, Imperial College, London, United Kingdom
71. Royal Brompton Hospital, London, United Kingdom
72. Department of Internal Medicine, Clinic University Hospital, INCLIVA Health Research Institute, Valencia, Spain
73. Department of Medicine, School of Medicine, University of Valencia, Valencia, Spai
74. Department of Intensive Care, Martini Hospital, Groningen, the Netherlands
75. Intensive Care Unit, Hospital do Espírito Santo, Évora, Portugal
76. Department of Cardiology, Northumbria Healthcare NHS Foundation Trust, Newcastle, United Kingdom
77. Benha Faculty of Medicine, Benha, Egypt
78. Department of Nursing, Tehran Heart Center, Cardiovascular Diseases Research Institute, Tehran University of Medical Sciences, Tehran, Iran
79. Barts National Institute for Health Research (NIHR) Biomedical Research Center, William Harvey Research Institute, Queen Mary University of London, United Kingdom
80. The Dutch Network for Cardiovascular Research (WCN), Utrecht, the Netherlands
81. Department of Cardiology, Amphia Hospital, the Netherlands
82. Department of Cardiology, Slingeland Hospital Doetinchem, the Netherlands
83. Department of Cardiovascular Research, Tehran Heart Center, Cardiovascular Diseases Research Institute, Tehran University of Medical Sciences, Tehran, Iran
84. National Institute for Health Research (NIHR) Exeter Clinical Research Facility, Royal Devon and Exeter Hospital and University of Exeter College of Medicine & Health, Exeter, United Kingdom
85. Department of Cardiology, Leiden University Medical Center, Leiden, the Netherlands
86. Julius Center for Health Sciences and Primary Care, University Medical Center Utrecht, Utrecht University, Utrecht, the Netherlands
87. Department of Cardiology, Maasstad Hospital, Rotterdam, the Netherlands
88. Department of Cardiology, Rijnstate Hospital, Arnhem, the Netherlands
89. Department of Cardiology, University Hospitals of Geneva, Geneva, Switzerland
90. Department of Cardiology, Martini Hospital, Groningen, the Netherlands
91. Department of Cardiology, Heart Center Hasselt, Jessa Hospital, Hasselt, Belgium
92. Department of Cardiology, Catharina Hospital, Eindhoven, the Netherlands
93. Department of Educational Development and Research in the Faculty of Health, Medicine and Life Sciences, Catharina Hospital, Eindhoven, the Netherlands
94. Department of Cardiology, Vrije Universiteit Amsterdam, Amsterdam Cardiovascular Sciences, Amsterdam, the Netherlands
95. Department of Cardiology, Dijklander Hospital, Hoorn, the Netherlands
96. Department of Intensive Care Medicine, Maasstad Hospital, Rotterdam, the Netherlands
97. Cardiovascular Research, Antwerp University and Cardiology, Antwerp University Hospital, Antwerp, Belgium
98. Department of Pulmonology, Medisch Spectrum Twente, Enschede, the Netherlands
99. Department of Intensive Care, Ziekenhuis Groep Twente (ZGT), Almelo, the Netherlands
100. Department of Cardiology, Zaans Medical Center, Zaandam, the Netherlands
101. Department of Internal Medicine, Antonius Hospital, Sneek, the Netherlands
102. Department of Cardiology, Bernhoven Hospital, Uden, the Netherlands
103. Department of Cardiology, Albert Schweitzer Hospital, Dordrecht, the Netherlands
104. Department of Cardiology, Rode Kruis Hospital, Beverwijk, the Netherlands
105. Department of Cardiology, Beatrix Hospital, Gorinchem, the Netherlands
106. Department of Cardiology, CHVZ, University Hospital Brussels, Jette, Belgium
107. National Institute for Health Research Biomedical Research Center, University College London Hospitals, London, United Kingdom
108. Department of Cardiology, Medical Center Leeuwarden (MCL), Leeuwarden, the Netherlands
109. Department of Cardiology, van Weel-Bethesda Hospital, Dirksland, the Netherlands
110. Department of Pulmonology, Ziekenhuis Groep Twente (ZGT), Almelo, the Netherlands
111. Freeman Hospital, Newcastle Upon Tyne NHS Hospitals Foundation Trust and Newcastle University, Newcastle Upon Tyne, NE7 7DN, United Kingdom
112. Department of Cardiology, St. Jansdal Hospital, Harderwijk, the Netherlands
