# Supplementary figures and images for: Electrocardiogram-based mortality prediction in patients with COVID-19 using machine learning
Source: Neth Heart J. 2022 Mar 17;30(6):312–8. doi: 10.1007/s12471-022-01670-2 (PMC8929464; doi:10.1007/s12471-022-01670-2)

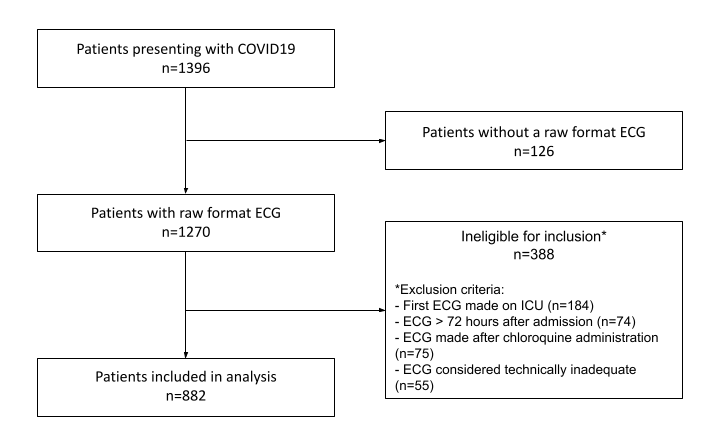

Supplement: Supplementary file 2 — Fig. S1 Flow chart of the patient selection process. (ECG electrocardiogram, ICU Intensive Care Unit) [file 12471_2022_1670_MOESM2_ESM.docx]

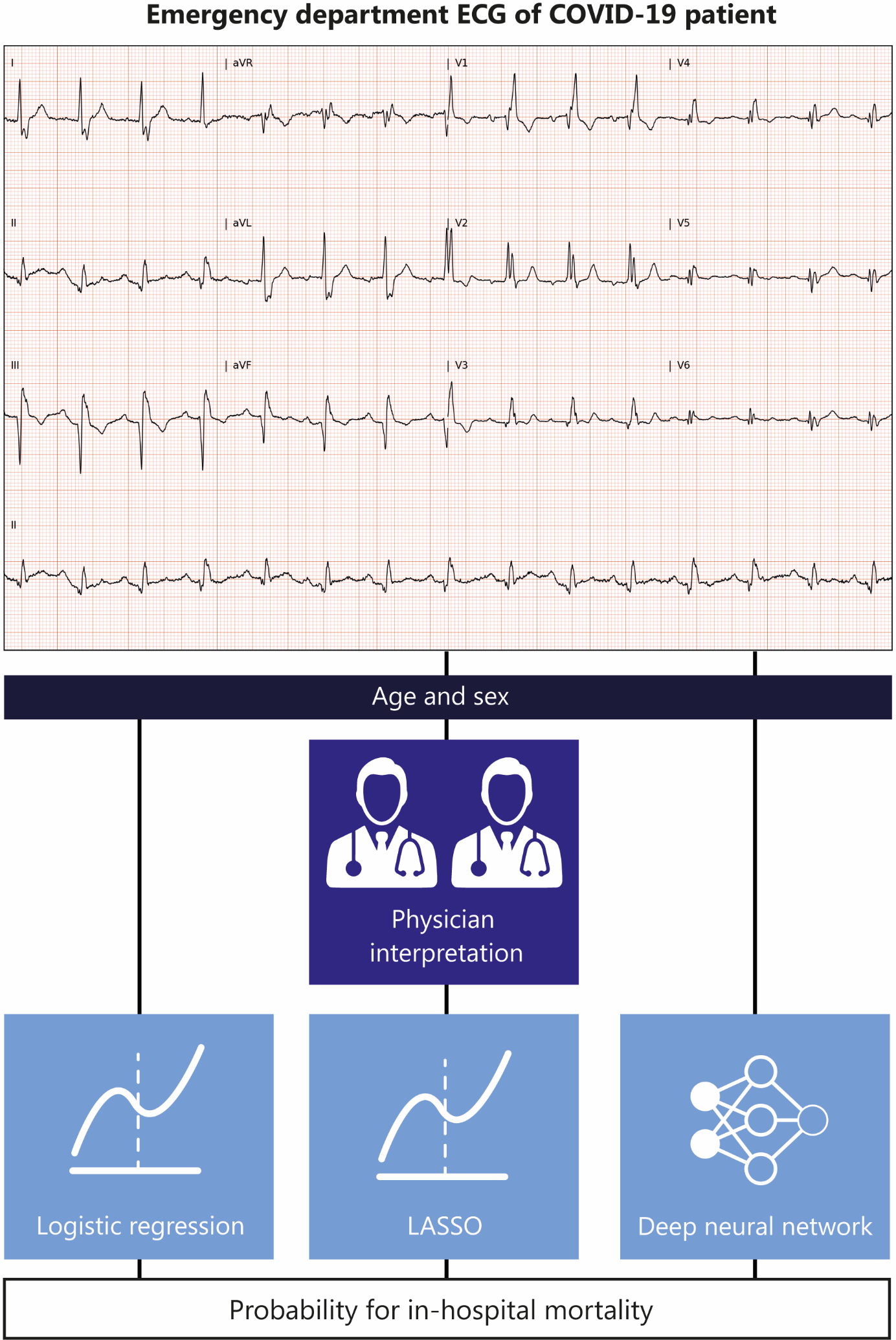

Supplement: Supplementary file 3 — Fig. S2 Overview of the two machine learning methods used. The LASSO approach consists of two steps, where the ECGs are (1) manually evaluated by a panel of physicians and (2) classified, while the deep neural networks takes raw ECGs as input and classifies in an end-to-end manner. This specific case had a high probability of in-hospital mortality. Follow-up of this case showed that the patient had died during admission. (ECG electrocardiogram, LASSO least absolute shrinkage and selection operator) [file 12471_2022_1670_MOESM3_ESM.docx]
